# HURMAT: an alternative approach for fatigue diagnosis and management based on ergonomics and human factors

**DOI:** 10.3389/fpubh.2026.1813438

**Published:** 2026-07-01

**Authors:** Tatiana Teixeira, Eduardo Carvalho, Maria Covas, Joana Guedes, Nilza Ramião

**Affiliations:** 1 INEGI - Instituto de Ciência e Inovação em Engenharia Mecânica e Engenharia Industrial, Porto, Portugal; 2 Associated Laboratory for Energy Transports and Aeronautics (PROA/LAETA), Faculty of Engineering, University of Porto, Porto, Portugal

**Keywords:** fatigue management, occupational fatigue, task performance, worker well-being, workplace ergonomics, human-centric approach occupational physiology

## Abstract

**Introduction:**

The increase in workplace accidents and the growing complexity of work environments have highlighted the need for more sustainable and adaptive occupational health and safety (OHS) management systems. Traditional approaches often fail to fully capture the variability of workers’ responses and the multifactorial nature of occupational risks.

**Methods:**

To address these challenges, an alternative methodology for the development of a human-centered risk management tool (HURMAT) is proposed. This methodology integrates the core principles of conventional OHS frameworks with a more in-depth, worker-centered evaluation of functional capacity. It is structured across different levels of a traditional OHS approach, allowing flexible implementation without requiring full deployment if detailed analysis is not needed. The approach incorporates occupational psychophysiology and considers work-related risk factors, enabling the diagnosis and management of fatigue.

**Results:**

The HURMAT methodology enables a more comprehensive characterization of tasks by integrating workers’ physiological characteristics and assessing physical, cardiovascular, and psychosocial loads. This approach supports a more detailed understanding of risk exposure and worker responses, improving the identification and management of occupational hazards.

**Discussion:**

HURMAT contributes to the modernization of OHS practices by promoting safer and more adaptive work environments. It underscores the need for a paradigm shift in how occupational risks are conceptualized and managed, emphasizing personalization, data-driven decision-making, and the recognition of individual differences. These elements represent fundamental pillars for fostering healthier, more inclusive workplaces, particularly in the context of ongoing organizational change.

## 1 Introduction

In 2022, an estimated 2.97 million non-fatal occupational accidents were reported in the European Union, each resulting in at least four calendar days of absence from work, in addition to 3,286 fatal accidents. It is noteworthy that the construction sector exhibited the highest prevalence of such incidents, followed sequentially by the sectors of transportation and storage, manufacturing, and agriculture, forestry, and fishing (1). In response to the growing incidence of workplace accidents in recent years, there has been an increasing national and global demand for OHS management systems that can be audited and implemented sustainably. The primary objective of these management systems is to streamline, clarify, and enhance OHS practices within the workplace. By fostering a more effective prevention system, these frameworks promote the active participation of all employees, regardless of hierarchical differences, in integrating safety measures into operational processes. Consequently, the implementation of such systems can lead to a reduction in workplace accidents and occupational diseases, support the advancement of scientific research in OHS, and contribute to the overall economic and social development of nations (2–4). The International Labor Organization (ILO) has also established guidelines for occupational safety and health management systems under ILO-OSH 2001. These guidelines are designed to protect workers, enhance their health status, and achieve higher standards of health and safety in the workplace. Additionally, they define clear responsibilities for the tripartite constituents, governments, employers, and workers, ensuring a structured and collaborative approach to occupational safety and health management (5, 6).

The automation of workplaces has introduced several socio-technical challenges, primarily due to the increasing control of work processes by machines, which has led to new health-related impacts on workers, particularly in terms of psychological well-being and fatigue, with the latter being a significant concern nowadays (7). Although there is no universally accepted definition of fatigue, the Canadian Centre for Occupational Health and Safety (CCOHS) describes it as a psychophysiological state resulting from insufficient sleep, prolonged mental or physical activity, or extended periods of stress or anxiety. Additional factors, such as repetitive tasks, may exacerbate the perception of fatigue. This state is typically characterized by symptoms including irritability, weariness, tiredness, reduced alertness, impaired concentration and memory, and physical exhaustion, among others. Several regulations and methodologies have been established to support the management of worker fatigue. For instance, Chander and Cavatorta (8) introduced the Postural Ergonomic Risk Assessment methodology, which is based on ISO 11226:2000 and EN 1005-4:2008 standards. Similarly, for repetitive work tasks, validated methodologies based on ISO 11228-3:2007 are available (9), aiming to reduce the incidence of musculoskeletal disorders. In addition, other observational methodologies can be applied, such as the Rapid Upper Limb Assessment (RULA), the Rapid Entire Body Assessment (REBA) and Ovako Working Posture Analyzing System (OWAS). However, these methods are often criticized for their limited resolution and sensitivity and often require adaptation to adequately capture task-specific ergonomic risks (10).

Comprehensive assessment of fatigue across its multiple dimensions may represent a valuable strategy for the prevention of occupational accidents and diseases, as well as for enhancing productivity, thereby contributing to safer and healthier workplaces (11). Monitoring and managing fatigue constitute a proactive approach to OHS that extends beyond the use of numerical indicators alone. This perspective emphasizes the need to integrate the work environment characteristics, the individual worker’s characteristics, and their psychophysiological responses to specific tasks. Such a multidimensional approach recognizes that fatigue is not merely the result of quantifiable workload or time-on-task measures but is also shaped by factors such as task complexity, work schedules, circadian rhythms, individual susceptibility, and recovery opportunities (12). By addressing these interrelated dimensions, fatigue management systems can provide more accurate assessments of worker capacity, support the prevention of human error, and reduce the likelihood of occupational accidents and illnesses (13, 14). Moreover, integrating individual and organizational factors into fatigue monitoring frameworks contributes to the development of safer, healthier, and more sustainable workplaces (12, 15).

Consequently, OSH management models must be re-evaluated to support the development of policies, planning strategies, risk control measures, and the assignment of responsibilities. Workplaces have undergone substantial transformations with the implementation of robotics and artificial intelligence (16). While these innovations may reduce certain pre-existing risks, they simultaneously introduce novel emerging risks that have the potential to increase fatigue and cause severe occupational accidents and diseases. Consequently, the emergence of these new risks renders traditional methodologies for occupational risk assessment insufficient, highlighting the need for updated approaches that account for psychosocial and cognitive factors (17).

Nevertheless, the introduction of new tools and innovative approaches to monitoring occupational risks faces significant challenges and resistance from both workers and safety managers. This resistance is largely attributed to a lack of knowledge, limited trust in these tools, and the initial investment associated with the required interventions (18). Some of the challenges encountered arise from the difficulty of developing OHS regulations at a pace that keeps up with workplace innovations. As a result, existing regulatory frameworks often prove inadequate to provide the necessary oversight and adaptation to evolving work environments (19).

Furthermore, OSH techniques must be reconsidered to accommodate the need for novel interventions that address emerging challenges and newly identified risks in modern work environments. Traditional occupational risk control methods are becoming insufficient, necessitating the adoption of approaches tailored to evolving workplace conditions (20–24). Indeed, these new approaches present socio-technical challenges in the development and implementation of programs aimed at monitoring workers’ health and effectively identifying and mitigating risks associated with environmental factors (25, 26).

**Figure 1:**
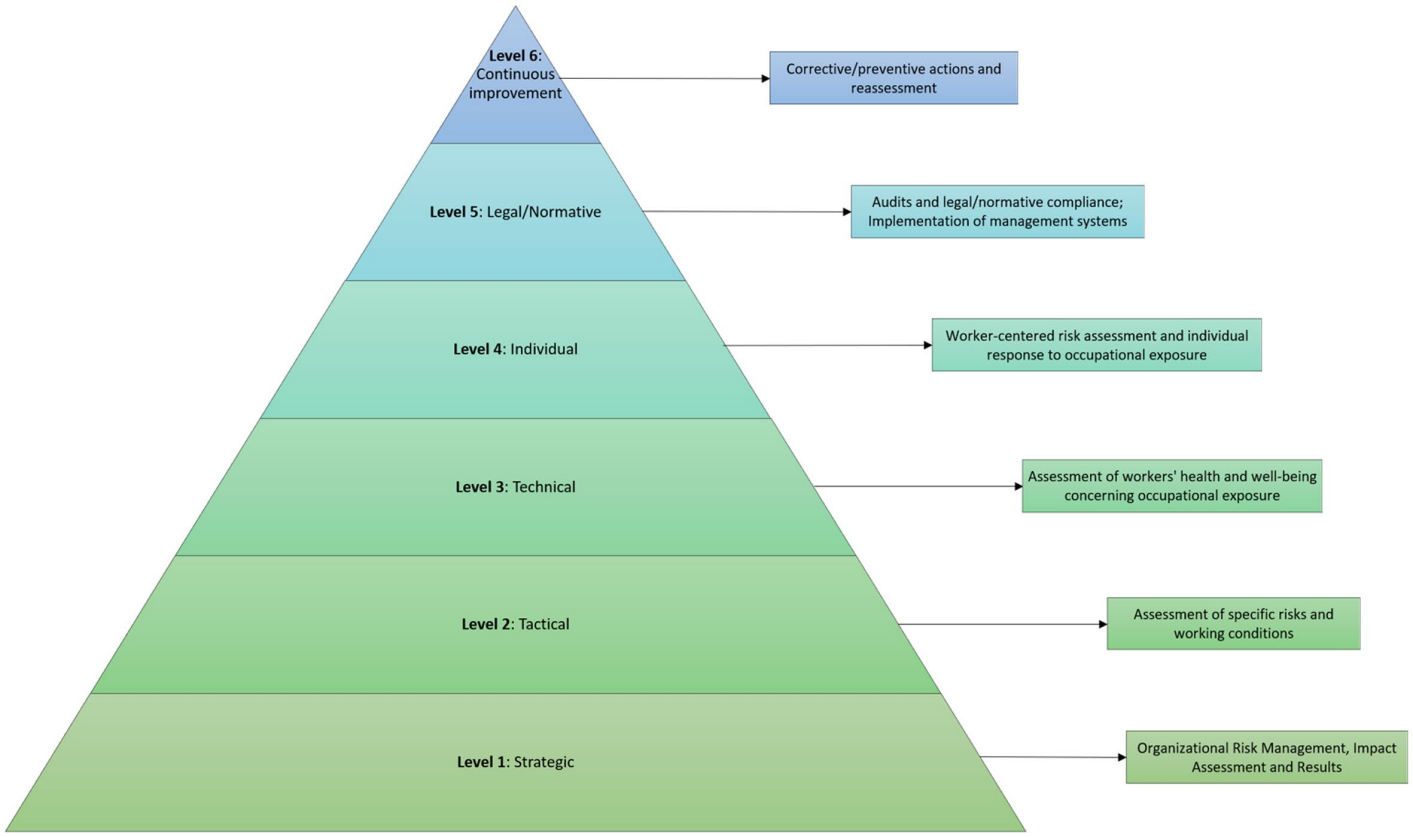
Different levels in OHS approaches – adapted from ISO 45001:2018 (52).

However, existing methodologies do not establish a direct link between task characteristics and the worker’s physiological response capacity, based on maximum work capacity. As a result, they do not ensure that tasks are performed under safe conditions that minimize the risk of injuries and occupational diseases. Figure 1 illustrates the novel approaches proposed that align with the evolution of modern workplaces.

The primary objective of this article is to propose a worker-centered methodology for diagnosis and management of fatigue, emphasizing workers’ response to occupational tasks from the perspective of occupational psychophysiology and considering the influence of multiple workplace risk factors. The proposed methodology aims to reassess OHS approaches by incorporating different intervention levels to more effectively address the emerging demands and challenges of modern workplaces. Based on the aim of this work, five research questions (RQ) were defined:

- RQ1—diagnostic dimension: To what extent do psychophysiological indicators provide greater accuracy in diagnosing occupational fatigue compared to traditional self-report and observational methods?
- RQ2—multilevel interventions: How effective are multilevel interventions in reducing fatigue-related incidents and improving OHS outcomes?
- RQ3—worker-centered approaches: How do interactions between environmental risk factors (e.g., noise, shift work, repetitive tasks) and individual worker characteristics (e.g., age, physical fitness, sleep quality) shape the onset and progression of occupational fatigue?
- RQ4—intervention and outcomes: What is the impact of implementing multilevel fatigue management interventions on reducing fatigue-related incidents in the workplace? Does systematic fatigue monitoring contribute not only to the prevention of occupational accidents and diseases but also to improvements in productivity and work quality?
- RQ5—technological integration: How can emerging technologies (e.g., wearable sensors, artificial intelligence, digital twins) be effectively integrated into fatigue monitoring and management systems to enable proactive and personalized interventions?

## 2 Approach and methodology

### 2.1 Framework overview

Identifying and managing risk variables during risk assessment is essential for designing and planning work activities that minimize injuries and enhance labor productivity. Various factors influence workers’ physical and mental well-being, including hygienic, anthropometric, physiological, psychophysiological, and psychological aspects, all of which should be considered in ergonomic risk assessments (3, 27, 28).

According to the European Working Conditions Survey carried out in 2021, 24% of the population surveyed in Portugal reported that their work involves performing tasks at a high pace, higher than the figure for Europe, where for the same category a response rate of 22% is presented, demonstrating that Portugal is 2% above the figure found in Europe (29, 30). These findings align with the definition of effort in an occupational context, where the term encompasses the dimensions of posture, direction, force intensity, and the persistence/duration of the activity, whether performed in a static or dynamic regime (31). These dynamics influence employees’ perception of well-being and exacerbate occupational health implications, increasing the risk of injuries and musculoskeletal disorders, among other conditions. The Human Factors approach can be categorized into four main groups: checklists, surveys, and reports; observation-based methods; direct measurement methods; and computer-based applications. Each category offers distinct approaches to evaluating work risks, ranging from qualitative assessments and expert observations to quantitative data collection and advanced computational analyses (28).

The implementation of non-invasive devices and mobile applications allows for real-time monitoring of workers’ physical and psychological conditions in different environments, facilitating dynamic adjustments to workloads and rest schedules based on individual needs (32–34). This integration yields substantial benefits, including increased productivity, reduced fatigue-related accidents, and lower absenteeism rates (35).

Furthermore, solutions such as Personnel Prognostics and Health Management provide predictive tools to assess health-related risks, particularly in high-risk professions, such as machine operators, drivers, and workers in hazardous environments, contributing to a safer and more efficient industrial workforce. The introduction of these technologies into the industrial sector also fosters a healthier and more efficient work environment while simultaneously reducing operational costs and improving the quality of work. Advancements in monitoring devices and applications can add significant value to the industrial sector by integrating physiological data into effort and productivity management, resulting in competitive advantages and improved quality of life for workers (36). While these techniques provide precise and objective data, their applicability is often limited due to the need for specialized equipment and technical expertise (37).

In line with these considerations, increased physical activity intensity leads to a higher constant state of oxygen use, contributing to the overall energy expenditure of the exercise. Beyond this threshold, glycolysis remains active and creates lactate build-up in the bloodstream, quickly intensifying fatigue. Such physiological responses have significant effects on the human body, especially on the cardiovascular system (38), further underscoring the importance of holistic strategies that address both the psychological and physical aspects of employee well-being. The integration of advanced technologies for physiological data monitoring has demonstrated significant potential in transforming the management of physical and mental effort in industrial environments. The continuous tracking of biomarkers, such as heart rate, blood pressure, and glucose levels, enables the early detection and prevention of health-related issues, thereby enhancing workplace safety and worker well-being. Originally developed for medical applications, these technologies are now being adapted for industrial use, offering a more personalized and data-driven approach to workforce management (36).

Posture-related tasks introduce extra hazards and significantly increase worker fatigue through the additional effort required to maintain static postures and sustain muscle forces. Repeated application of these forces, particularly when they exceed the recommended levels of muscular force, can elevate the risk of work-related injuries (39). It is important to note that physically demanding tasks such as those involving high-intensity loads that demand increased muscle contractions, combined with the worker’s adaptation to the work environment, can create a physiological imbalance. This imbalance stems from the increased production of lactic acid, which raises blood acidity and intensifies cardiovascular stress. Workers with lower physical and cardiorespiratory fitness are more susceptible to overload (40–42). Given that heart rate regulation shifts from the parasympathetic system during rest to the sympathetic system during physical exertion, it’s anticipated that low-frequency values will increase throughout the workday due to the physical demands of the job. Conversely, to maintain equilibrium between the sympathetic and parasympathetic systems of the body, that the high-frequency (HF) values are also expected to rise during the day (43, 44). The HF band, representing the respiratory band, reflects heart rate variability associated to the respiratory cycle (45). As this band is primarily regulated by the parasympathetic nervous system, reduced HF power at specific times of the day may indicate tasks that elevate worker stress and anxiety, thereby raising their cardiovascular load (46).

Effective management of work and recovery periods is essential if workers are to maintain their sympathovagal balance. Effort management programs based on the worker’s physiological response to the task hold the key to improving working conditions and productivity, and can significantly reduce a worker’s cardiovascular load by around 30% (47). In addition, these tools can represent a management indicator that allows workers to be rotated to less physically demanding tasks, as well as identify the breaks needed for worker recovery (48–51). The methodology presented addresses the challenges companies face in providing favorable working conditions to mitigate musculoskeletal injuries associated with manual load handling and cardiovascular diseases associated with long-term exposure to physically demanding work. These injuries remain a significant source of absenteeism, thereby increasing operational costs and reducing productivity.

Most ergonomic tools incorporate only basic worker characteristics, such as age and gender, without accounting for the broader physiological differences that influence individual responses to physical workload. However, integrating workers’ physiological characteristics into the assessment process offers a more comprehensive understanding of task demands and the associated physical, cardiovascular, and psychosocial loads. This more holistic perspective appears to be a critical step toward advancing OSH management systems.

### 2.2 Methodology for the development of a human-centered risk management tool (HURMAT)

The proposed methodology is worker-centered, focusing on the worker’s physiological capacity to meet job demands. To achieve this, the methodology is structured according to different levels of an OHS approach, based on ISO 45001:2018 (52) and ISO 31000:2018 (53). The different levels were designed and structured to address the challenges identified in the workplace and to support OHS teams in overcoming difficulties related to the implementation of legislation, as well as in preventing occupational accidents and diseases observed among workers. Each level has a specific objective and is intended to address specific focal points that support the analysis of critical aspects related to the identified problem and the identification of the most appropriate solutions. It is important to highlight that the proposed methodology does not need to be implemented as a whole and may be concluded at an earlier stage if there is no interest in conducting a detailed analysis of workers’ responses. Additionally, OHS teams may decide to terminate the study at any stage. However, it is essential to emphasize that the success of the methodology depends on its correct and consistent implementation, as well as the active participation of OHS teams, workers, direct supervisors, and company management.

#### 2.2.1 Level 1: initial diagnosis of workstation issues

This level aims to assess the extent of the issues identified in the workplace. This initial phase of the methodology is crucial for defining the various approaches and techniques that should be considered. The successful implementation of this methodology requires active engagement and collaboration among all stakeholders. Before applying the proposed methodology, several key scientific questions (KSQ) should be addressed to guide the study toward the OHS issue and ensure the collection of the most relevant information:

- KSQ1: Have there been previous attempts to resolve the identified issue using other approaches? If so, which ones?
- KSQ2: Have any improvements been observed with previous solutions? If so, what were they?
- KSQ3: What are the primary concerns and complaints of workers?
- KSQ4: Why do you believe traditional approaches have failed to resolve the problem? (e.g., automation of workplaces, lack of worker acceptance, etc.).
- KSQ5: How many workers are affected? Which tasks raise the greatest concern?

#### 2.2.2 Level 2: observation and identification of occupational exposure

Level 2 should involve the initial diagnosis of issues identified at the workstation. This process should be carried out through comprehensive work analyses. This approach should facilitate an in-depth evaluation of the workstations by examining work methods, task objectives, productivity, and associated risks.

- KSQ1: What are the main risks associated with the tasks?
- KSQ2: Are there indicators of physical fatigue (e.g., manual handling, pushing and pulling, etc.)?
- KSQ3: Are workers exposed to extreme environments?
- KSQ4: Are the tasks automated in any way?
- KSQ5: Are the tasks performed by a group of workers with specific characteristics?
- KSQ6: What are the workers’ daily goals? Are there any incentives or rewards associated with their performance?

#### 2.2.3 Level 3: application of tools for monitoring workers’ health status

This evaluation level must be conducted following ethical standards and occupational medicine guidelines to ensure the proper monitoring of workers’ health status. A comprehensive understanding of the workstation should be achieved by collecting demographic data, symptom analysis, and clinical and professional history through surveys and interviews. This integrated approach enables a thorough assessment of the work environment, establishing a solid foundation for the development of targeted and effective interventions.

- KSQ1: What are the main complaints reported by the workers?
- KSQ2: What are the primary symptoms identified?
- KSQ3: Are there any existing occupational diseases associated with the tasks?
- KSQ4: What are the main difficulties experienced by workers in performing their tasks?
- KSQ5: Have workers suggested any improvements?

#### 2.2.4 Level 4: real-time and continuous assessment of the workers’ physiological response

At this methodological stage, the main objective is to use physiological indicators to determine the level of worker fatigue in response to task demands. Fatigue can be classified into physical, cardiovascular, and cognitive/psychological categories. Its assessment should be conducted alongside a detailed analysis of occupational risks and environmental exposure conditions. At this level, there are no specific RQs to be addressed. The focus is on collecting psychophysiological data based on the variables identified in the previous levels. This stage represents the midpoint of the study, serving to gather information highlighted in earlier phases. Progression to subsequent stages depends on the successful completion of this phase.

- KSQ1: Is it possible to combine objective and subjective methodologies?
- KSQ2: Is it feasible to integrate methodologies involving multiple instruments?
- KSQ3: Can methodologies and equipment be aligned with the workstation and the task?
- KSQ4: Can the instruments and methodologies affect work comfort?

#### 2.2.5 Level 5: comparison with reference values to identify safe health indicators for workers

The objective of this methodological level is to establish guidelines that justify the physiological responses to the workload to which workers are exposed. This stage aims to provide a scientific foundation for interpreting physiological indicators in the context of occupational workload, ensuring that assessments are both reliable and applicable to real-world work conditions. At this stage, it is crucial to address key scientific questions that are fundamental for an accurate occupational health risk assessment:

- KSQ1: Is the worker’s physiological response truly representative of the task’s demands, or is it merely an atypical reaction influenced by individual characteristics?
- KSQ2: Should physiological values be analyzed as generalized health-normalized reference values, or should they be context-specific?
- KSQ3: Are there existing guidelines that define standardized physiological values for occupational settings like those under investigation?
- KSQ4: What are the critical points identified in the workers’ physiological responses?
- KSQ5: What is the rationale behind the observed physiological changes?

#### 2.2.6 Level 6: implementation and evaluation of intervention measures and monitoring

The increasing complexity of occupational environments underscores the necessity of methodologies rooted in physiological approaches and tailored to individual characteristics. Such methodologies aim to enhance workplace adaptation and product development. The final stage of the proposed methodology involves supporting the implementation of solutions and identifying decision-support indicators. This process is carried out in close collaboration with company management and operational leadership, ensuring alignment with organizational goals. This level does not aim to address specific scientific questions, as it depends on the results of the overall process and the implementation of strategies by the companies.

- KSQ1: Are the measures applicable to the workstation and the task?
- KSQ2: Are the measures effective and do they address the entire problem identified during the diagnostic phase?
- KSQ3: Is there follow-up and monitoring of the implemented measures?
- KSQ4: Has the risk level been reduced?

## 3 Results

**Figure 2:**
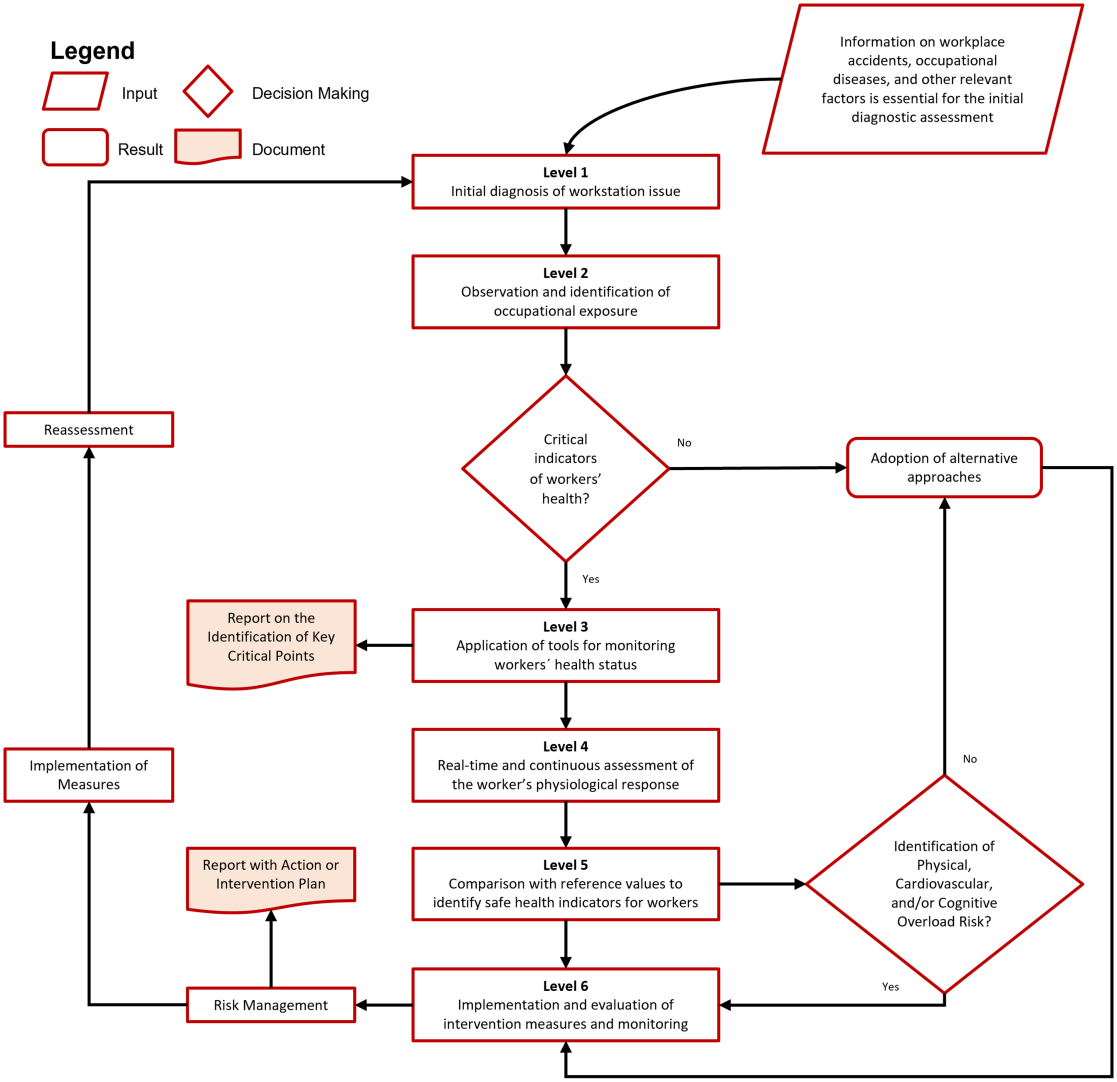
Worker human-centered risk management tool stages.

This section presents the procedures that should be adopted for the implementation of the HURMAT methodology. Figure 2 illustrates the different stages of the proposed solution for analysing physiological and productivity metrics. Throughout the methodological process, the development of various reports is suggested as an indicator of stage completion. These reports support a strategy of active corporate engagement in methodological monitoring and decision-making processes, contributing to the formulation of improvement strategies and the evaluation of their quality and effectiveness.

### 3.1 Level 1: initial diagnosis of workstation issues

After addressing the RQs outlined, all pre-existing documentation should be analyzed to guide the subsequent steps of the study. All data relevant to this initial phase must be provided by the company where the study should be conducted. These data may include:

- Workplace accident records (54);
- Accident investigation reports (55);
- Most affected body regions as reported by workers (56);
- General and specific risk assessments previously conducted by the company’s OHS team (57);
- Absenteeism rates and corresponding causes (58);
- Reported occupational diseases (59);
- Other relevant information.

Level 1 serves as the strategic foundation for decision-making regarding the most effective tools to address the identified issue. To achieve this, the problem must be examined by a multidisciplinary team capable of approaching it from diverse perspectives. Furthermore, Level 1 is essential for the implementation of all subsequent stages, functioning as a framework level that ensures the continuity of the OHS intervention pyramid rather than a level focused on achieving immediate, objective results. This level is critical, as it involves the collection of comprehensive information regarding the current state of OHS within organizations and the identification of intervention needs. It serves as the input for determining the appropriate tools and methodologies to be applied in subsequent levels.

### 3.2 Level 2: observation and identification of occupational exposure

During this phase, workstation assessments should be carried out through direct observation, supported by photographs and video recordings to document key tasks and activities (60). To account for variability in task execution each task should be observed three times, with different employees performing each iteration. The identification of critical indicators—those objectively responsible for negative health impacts on workers—serves as a key decision point within the proposed methodology. If the identified indicators can be effectively addressed using traditional OHS approaches, the methodology may conclude at this stage. However, if traditional approaches prove insufficient, the process should continue, exploring alternative, worker-centered solutions. Moreover, at this decision-making level, the identification of critical points allows for the confirmation of needs that will serve as input for Level 3, where the appropriate tools are selected for application across the various workstations.

### 3.3 Level 3: application of tools for monitoring workers’ health status

Data collection should be conducted through interviews and the use of questionnaires and/or scales that allow for the gathering of information related to the worker’s clinical and professional history to identify potential correlations (61). These tools help to systematically capture relevant data and provide valuable insights into the workers’ health and work-related conditions. Other tools may be essential for the application to identify key points that lead to potential solutions for the identified issue. These additional tools can provide further insights into the underlying factors affecting workers’ health and productivity, enabling a more comprehensive understanding of the problem. The use of such tools facilitates the detection of patterns, correlations, and risk factors, which are crucial for developing targeted interventions and effective solutions. Based on the results obtained at this level, it becomes evident which tools and variables are essential to be objectively and continuously monitored at the workstations.

### 3.4 Level 4: real-time and continuous assessment of the workers´ physiological response

At this level, data collection employs advanced detection methodologies to gather comprehensive physiological information. Real-time monitoring plays a crucial role in recognizing critical occupational contexts, enabling the assessment of a worker’s capacity to perform tasks based on physiological indicators and biomarkers.

For a holistic evaluation, additional physical, cardiovascular, and cognitive parameters can be integrated, enhancing the precision of occupational fatigue analysis. The correlation between physiological data and performance indicators allows for the identification of inadequate workload management and other factors that may negatively impact worker health and organizational efficiency. This approach facilitates the personalization and optimization of work activities, aiming to mitigate fatigue and, consequently, promote worker safety, health, and overall well-being (62).

#### 3.4.1 Physical fatigue

Physical fatigue is directly related to the biomechanical effort required to perform a task and may be influenced by several factors, including manual handling of loads, improper postures, repetitive work, and prolonged muscle exertion. Data collection for this evaluation may include indicators such as maximum aerobic capacity (VO_2_ max), lactate levels, physiological load, and muscle-specific variables such as glycogen depletion (63). These variables can be monitored alongside kinematic and kinetic movement analysis, enabling a comprehensive assessment of metabolic expenditure and the muscular recruitment required for work tasks (64).

Furthermore, exposure to adverse environmental conditions, such as extreme temperatures, can exacerbate physical fatigue. Monitoring skin and core temperatures, combined with cardiovascular and metabolic variables, provides a more precise assessment of how environmental factors impact worker endurance (65). This information can guide intervention strategies such as implementing rest periods or task rotation, ensuring that workplace conditions are tailored to minimize excessive fatigue and prevent musculoskeletal injuries or long-term occupational diseases (66).

#### 3.4.2 Cardiovascular fatigue

Cardiovascular fatigue results from the excessive strain placed on the circulatory system during occupational activities, particularly those involving high physical intensity or performed in extreme environmental conditions. This type of fatigue can be assessed through physiological biomarkers such as heart rate, heart rate variability, and blood pressure.

The integration of these measurements with physical performance indicators enables the identification of cardiovascular overload, ensuring that task demands are aligned with the worker’s capacity. For example, prolonged exposure to hot and humid environments can elicit intense physiological responses, increase heart rate and reducing circulatory efficiency, which may compromise both worker safety and performance (67).

Other critical parameters may also contribute to overall worker fatigue, often associated with rotating shift work, high noise levels, or additional environmental stressors. To evaluate these impacts, the methodology proposed here includes objective sleep quality assessments and rest period monitoring, which can help confirm the negative effects of irregular work schedules on worker health and performance.

#### 3.4.3 Cognitive and psychological fatigue

Cognitive and psychological fatigue is linked to mental exhaustion caused by intellectual workload, complex decision-making, and prolonged exposure to occupational stressors such as excessive noise, vibrations, inadequate lighting, and psychological pressure. This type of fatigue can be evaluated using physiological indicators, including heart rate variability, skin conductance, and brain activity patterns, along with neuropsychological tests and subjective fatigue scales.

Cognitive fatigue can impair concentration, reaction time, and decision-making abilities, increasing the risk of operational errors and workplace accidents. Continuous monitoring of these parameters allows for task organization adjustments, preventing performance decline and ensuring greater worker well-being. However, real-time monitoring of cognitive fatigue presents greater challenges due to the complexity of occupational environments (68).

Nevertheless, whenever possible and necessary, this methodology recommends incorporating cognitive fatigue assessments, as long-term exposure to excessive mental workload and occupational stress may contribute to conditions such as depression and burnout. Addressing these risks proactively through appropriate workload management and workplace interventions is crucial for maintaining both worker health and productivity (69).

#### 3.4.4 Performance and productivity

Performance indicators should be monitored using internal data provided by each company. This information includes key variables such as load type (weight and stability), number of handling operations, lifting height, transport distance, availability of mechanical assistance, and working conditions (temperature and humidity). The collected performance indicator data should be analyzed to establish a comprehensive profile of load handling patterns and assess worker overload, facilitating the development of targeted interventions to optimize occupational safety and efficiency (70).

### 3.5 Level 5: comparison with reference values to identify safe health indicators for workers

Several established guidelines facilitate the analysis of monitored physiological values. For instance, the National Institute of Occupational Safety and Health (NIOSH) provides recommendations on maximum aerobic capacity (VO_2_ max) for work under normal and extreme environmental conditions (71), as well as guidelines for metabolic consumption estimation (72). However, these guidelines do not always sufficiently account for work-induced fatigue, nor do they offer comprehensive alternatives for correcting and improving workplace conditions.

Therefore, depending on the data collected, it is recommended to use scientifically validated reference values that are reliable and applicable in occupational settings, while also considering internationally recognized standards. Additionally, reference values from validated scales, questionnaires, and monitoring techniques should be consulted, provided they are scientifically justified and deemed appropriate for comparison with the collected data. This approach ensures that fatigue assessments are both evidence-based and adaptable, enabling more accurate evaluations of workload-induced physiological strain.

### 3.6 Level 6: implementation and evaluation of intervention measures and monitoring

Continuous improvement is a cornerstone of this approach, achieved through ongoing monitoring and adjustments tailored to the evolving needs and challenges of companies in implementing solutions. The indicators defined within this methodology are designed to align with the monitoring parameters established in earlier stages. Their primary objective is to guide OHS teams, as well as company management, toward adopting solutions that are customized to the company, the workstation, and, most critically, the workers.

These solutions must be detailed enough to ensure effective application and efficient workload management by employees. Proper design and implementation of these solutions can significantly reduce absenteeism, workplace accidents, and occupational diseases, and increase productivity and better company results. Customized solutions tailored to the realities of individual companies are pivotal for improving workplace conditions, increasing worker satisfaction, and reducing costs associated with absenteeism. The focus on the worker remains central to the success of this tool, fostering active collaboration between management teams and employees to enhance receptivity to new solutions.

This methodology emphasizes the critical role of worker-focused approaches, promoting the development of adaptive solutions that benefit both employees and organizations. By integrating physiological insights with individualized strategies, it ensures a safer, healthier, and more productive work environment.

Equally important is the continuous monitoring of the implemented solutions to verify whether they are effectively addressing the identified challenges. Regular reassessment of these solutions is essential to determine their actual impact and effectiveness. If the desired outcomes are not achieved, adjustments must be made, or alternative strategies should be considered. This iterative evaluation process not only ensures the sustainability of improvements but also provides critical feedback for refining future interventions. Assessing the efficacy of these solutions is a crucial step in understanding their quality and mitigating risk factors (73).

## 4 Practical implementation

In this section, a practical example of the proposed fatiguemanagement methodology is presented to illustrate its application in a realworld operational context. The objective is to demonstrate the applicability and validity of the methodology. The case study was conducted in a company from the retail industry employing more than 1,000 workers, focusing specifically on employees performing picking tasks within a highdemand logistics environment.

### 4.1 Level 1: initial diagnosis of workstation issues

The analyzed company exhibited a high absenteeism rate, primarily attributed to musculoskeletal disorders, as well as frequent complaints to occupational health services regarding pain or discomfort in various body regions, with the lower back being the most prevalent (KSQ3). The OSH team applied various ergonomic assessment tools, including RULA, REBA, and OWAS, all of which indicated a high level of risk associated with the picking task (KSQ5: this task emerged as the main source of concern, involving a significant portion of the workforce).

Ergonomic analyses prompted the OSH team to intervene by automating certain workstations, including the installation of pallet conveyor belts and vacuum lifting arms to assist with the handling of heavier boxes (KSQ1). Furthermore, workers with pre-existing musculoskeletal complaints were rotated to less physically demanding tasks. Despite these interventions, absenteeism rates did not improve (KSQ2). The vacuum lifting arm proved insufficient to meet all operational needs (KSQ4), particularly in cold environments where boxes are prone to tearing, thereby preventing effective use of the device.

### 4.2 Level 2: observation and identification of occupational exposure

Within its operational environment, several tasks involve manual material handling, with loads varying in size, weight, stability, and types of grips, some of which are suboptimal (KSQ2). Workers are also exposed to cold environments, including areas maintained at 0–4 °C and −24 °C (KSQ3). The picking task is considered the most critical, where workers (male and female, aged 19–66 years) are responsible for assembling pallets to fulfil customer orders (KSQ5). Pallet assembly is guided through a voice-pick system that instructs workers on which items and the number of boxes to place on each pallet. Additionally, workers move through multiple storage aisles using pallet trucks to complete the pallet assembly process.

The work analysis tool by T. Armstrong (74) was used to verify that the loads handled ranged from a few grams up to 25 kg. All loads involved movements that could include trunk twisting/tilting and arm elevation, depending on both the initial height of the box and the height at which it was placed. It was observed that, due to the pressure exerted on workers to maintain or increase productivity, they often carried more than one box at a time (KSQ6). In addition, the boxes could be unstable depending on the type of product stored. It was identified that the handling of liquid-containing boxes caused the greatest discomfort among workers (KSQ1).

### 4.3 Level 3: application of tools for monitoring workers’ health status

Interviews were conducted with the workers, and assessment tools were also employed, including the Nordic Musculoskeletal Questionnaire. Through this questionnaire, it was found that all workers already exhibited symptoms of pain or discomfort in regions such as the lower back, neck, and shoulders (KSQ2).

During the interviews, workers reported complaints associated with physical fatigue, manifested by symptoms such as muscular pain, headaches, a sensation of weakness, and decreased productivity, among others (KSQ3). Furthermore, participants expressed a low level of job satisfaction and perceived the picking activity as highly demanding from a physical perspective, as evidenced by the application of the Borg Scale (KSQ1 and KSQ4). All the workers interviewed suggested automating tasks whenever possible but also reducing the weights of the boxes handled (KSQ5).

### 4.4 Level 4: real-time and continuous assessment of the worker’s physiological response

Considering the information collected during the initial phase of the methodology, and given that physical fatigue was identified as the main focus of the problem in the industry under analysis, the following physical parameters were defined to assess the risks to workers’ health and safety (KSQ1, KSQ2 and KSQ4):

1. Assessment of physical workload: Application of accelerometery techniques to evaluate distances covered, energy expenditure, and metabolic rates. All data were correlated with productivity indicators provided by company management to identify critical loads, uninterrupted working times, and recommended rest periods.
2. Assessment of cardiovascular workload: Use of heart rate monitors to track the cardiovascular response to physical effort, to determine whether the reaction of the cardiovascular system was close to or exceeded the worker’s recovery capacity, based on their maximum aerobic capacity.
3. Assessment of perceived exertion: Identification of the relationship between objective data and the worker’s subjective perception of effort regarding the task performed.

All data were collected in real-time on the shop floor during the workers’ daily activities. Each worker underwent two stages of evaluation: a daily assessment (eight consecutive working hours) and a weekly assessment (covering a full workweek, including days off, to monitor cumulative fatigue) (KSQ3).

### 4.5 Level 5: comparison with reference values to identify safe health

The results obtained were evaluated in accordance with reference values established by the manual handling of loads standards (75–77) (KSQ1). These findings were further examined by a multidisciplinary team, including occupational physicians, who assessed the workers’ physiological responses (KSQ2 and KSQ3). The analysis suggested that the combination of physical exertion and exposure to low temperatures may trigger cardiovascular events, which over time could increase the risk of developing heart diseases. These observations were consistent with existing evidence highlighting the impact of occupational workload and environmental stressors on cardiovascular health (KSQ4 and KSQ5).

### 4.6 Level 6: implementation and evaluation of intervention measures and monitoring

Based on the results obtained, a set of interventions was proposed, tailored to the specific workstation and grounded in the workers’ physiological responses. The key interventions include (KSQ2):

1. Effort Management Tool: A tool specifically developed to categorize task-related risk according to the workers’ physiological responses. Designed exclusively for the evaluated workstation, this tool can manage worker exertion based on individual characteristics, the properties of the loads handled, and environmental conditions such as temperature and humidity.
2. Task Rotation Program: A structured program of task rotation aimed at reducing cumulative fatigue, implemented in conjunction with the Effort Management Tool to ensure alignment with physiological risk assessments.
3. Implementation of Complementary Medical Examinations: A set of supplementary medical tests was recommended for employees during recruitment to enable continuous monitoring from the outset of task execution and to prevent potential health risks. The data obtained from these examinations served as input for both the Effort Management Tool and the Task Rotation Program.

The implementation of the recommendations was the responsibility of the company’s OHS team, which agreed with all the recommendations provided (KSQ1). To ensure the effectiveness of the proposed measures in mitigating occupational fatigue, the implementation process was systematically monitored and followed throughout its execution (KSQ3).

The implementation of all measures was also monitored and supervised by the research team through productivity and fatigue indicators, in order to assess the effectiveness of the proposed interventions (KSQ4).

In the presented scenario, a 26% reduction in work-related musculoskeletal injuries was confirmed as a result of implementing the effort management tool. In addition, a 34% improvement in the Physical Performance Index was observed, indicating enhanced physical capacity following the intervention. The task rotation program allowed workers to recover from the most demanding tasks and led to a 92% improvement in pain levels, corresponding to an improvement of at least one level on the pain scale. Participant satisfaction with the ergonomic sessions also reached 95%, suggesting a high level of worker acceptance and engagement with the implemented measures.

The implementation of complementary medical examinations, together with all other suggested and implemented measures, resulted in a 46% reduction in diagnosed medical restrictions related to work-related injuries. Overall, these results demonstrate that the HURMAT methodology provided effective inputs for reducing pain and musculoskeletal injuries, as well as improving workers’ quality of life.

## 5 Discussion

### 5.1 Diagnostic dimension

Novel OHS approaches facilitate the development of interventions tailored to specific organizational contexts and based on the characteristics of individual workers. In an era marked by an aging workforce and growing difficulties in adapting to new roles, it is essential to meet workers’ specific needs to ensure their overall well-being. Fundamentally, OHS principles stipulate that work should be adapted to the worker, not the other way around (5). As it becomes increasingly difficult to uphold this principle, addressing individual needs has become paramount. The development and integration of new work methodologies are increasingly recognized as a necessity for OHS teams, aiming to promote a proactive stance in identifying and mitigating occupational risks, thereby ensuring worker well-being and reducing costs associated with workplace accidents and occupational diseases (78). Given these challenges, there is a growing consensus that OHS strategies must evolve from reactive models, often limited to retrospective incident analysis, to proactive systems that integrate real-time data, predictive indicators, and individualized risk profiling.

In recent years, OHS teams have faced significant challenges due to the constant transformation of workplaces. The increasing implementation of innovation and automation in workstations has rendered traditional methodologies increasingly insufficient in addressing the protection of workers exposed to emerging risks. Automated systems now dictate work rhythms and rest periods, contributing to increased worker fatigue and dissatisfaction with workplace conditions (79, 80).

The proposed HURMAT methodology introduces a worker centered approach that integrates psychophysiological indicators with task characteristics and work environment conditions, enabling a more comprehensive and objective assessment of occupational fatigue. In this regard, and in response to RQ1, the findings suggest that the application of psychophysiological indicators in the analysis of working conditions provides a higher level of diagnostic accuracy when compared to traditional methods based exclusively on self report and observational techniques.

Traditional assessment methods rely predominantly on the worker’s subjective perception and the observer’s interpretation and are therefore susceptible to cognitive and social biases. In contrast, psychophysiological indicators allow for the continuous and objective capture of physiological responses associated with fatigue, directly linked to task execution and contextual work conditions. This capability is particularly relevant for the early detection of fatigue states and for identifying interindividual variability that may not be verbalized or externally observable yet remains crucial for the prevention of occupational accidents and work-related diseases.

Previous studies have already highlighted the importance of monitoring physiological indicators to better understand worker–environment interactions and to identify physiological responses to work-related stress. Within this framework, the HURMAT methodology contributes to advancing occupational fatigue assessment by supporting a multidimensional and evidence based diagnostic approach that complements and enhances traditional assessment methods (81, 82).

Thus, the HURMAT methodology is shown to go beyond traditional approaches, not by replacing them, but by complementing them, thereby providing a more robust basis for the diagnosis of occupational fatigue in real-world work contexts. However, it should be emphasized that the interpretation of psychophysiological indicators must be framed within the specific context of the task and the work environment, reinforcing the importance of an integrated and multidimensional assessment approach.

### 5.2 Multilevel interventions

This study proposes an enhancement of OHS strategies by considering both the characteristics of workstations and the individual profiles of workers. The HURMAT is developed in alignment with the methodological foundations of ISO 45001:2018 (52) and ISO 31000:2018 (53).

Multilevel interventions play a critical role in addressing occupational fatigue by simultaneously targeting individual, task related and environmental determinants. In response to RQ2, the proposed HURMAT methodology demonstrates the effectiveness of a multilevel and system based approach in reducing fatigue related risks and improving OHS outcomes. This approach is particularly aligned with the emerging needs of increasingly digitalized work environments, where workers are exposed to imminent and complex risks that are often difficult to identify and mitigate, yet may have substantial impacts on health, safety and performance (81, 83).

The proposed framework enables the identification of the complex nature of occupational fatigue through the human–task–environment system, rather than relying solely on individual level analyses. By integrating worker centered psychophysiological data with contextual information related to task demands and work environment conditions, the HURMAT methodology supports preventive actions aimed at reducing occupational accidents and work-related diseases. Importantly, these interventions are adapted to the individual worker while remaining contextualized to a specific task and workplace conditions, thereby reinforcing their practical relevance and applicability in real-world settings.

While some authors advocate for more individualized approaches that address risks in an isolated manner, the HURMAT methodology adopts a multidisciplinary and integrative perspective. This multidimensional assessment enables the definition of preventive, improvement or mitigation measures that simultaneously address individual, organizational and environmental factors, overcoming the limitations of fragmented risk management strategies (84, 85).

The results obtained indicate that the HURMAT methodology effectively supports decision-making processes and contributes to a measurable reduction in empirical fatigue indicators. As demonstrated in the presented case study, the implementation of multilevel interventions guided by HURMAT was associated with a 26% reduction in work-related musculoskeletal injuries, a 46% decrease in self-reported worker pain, and a 60% increase in performance index. These outcomes highlight the potential of multilevel, data driven interventions not only to mitigate fatigue related risks, but also to positively influence both health related and productivity related OHS indicators.

While direct causality between the application of the methodology and long-term accident reduction requires further longitudinal investigation, the findings provide strong evidence that multilevel interventions supported by integrated assessment frameworks such as HURMAT can enhance fatigue management strategies, promote proactive risk prevention, and contribute meaningfully to improved occupational health and safety outcomes.

### 5.3 Worker-centered approaches

The holistic and worker-centered approach offered by the HURMAT methodology, which integrates physiological and cognitive variables with environmental, task-related and productivity indicators, provides a robust framework to analyse how interactions between workplace risk factors and individual worker characteristics shape the onset and progression of occupational fatigue. These interactions are critical, as exposure to environmental stressors such as noise, repetitive tasks or shift work does not elicit uniform fatigue responses, but rather depends on individual characteristics including age, physical fitness, sleep quality and functional capacity.

By continuously monitoring psychophysiological responses within the human–task–environment system, HURMAT enables the identification of situations in which environmental demands exceed individual adaptive capacity, thereby accelerating the onset of fatigue and contributing to its cumulative progression over time. This interaction helps to explain why fatigue may emerge earlier or intensify more rapidly in certain workers exposed to similar external conditions, highlighting the limitations of standardized or purely observational risk assessment approaches (79, 80).

In the presented case study, one of the key measures proposed to address this interaction was the implementation of an effort management tool that supported dynamic worker rotation and the adjustment of work–rest cycles based on monitored fatigue levels and individual worker characteristics. This tool enabled task allocation and recovery periods to be adapted in real time according to each worker’s physiological response and capability, thereby operationalizing a truly worker-centered intervention strategy. Such an approach directly illustrates how the interaction between environmental demands and individual variability shapes fatigue trajectories and how these trajectories can be effectively managed through adaptive organizational measures (86).

Furthermore, the integration of physiological metrics with task-specific and environmental risk parameters allows for a contextualized and dynamic evaluation process. This supports timely decision-making, optimization of work–rest schedules and task rotation strategies, reducing reliance on subjective judgments by supervisors or OHS technicians. As a result, the methodology facilitates early detection of fatigue-related conditions that may evolve into severe workplace accidents or long-term work-related diseases, while simultaneously contributing to improvements in productivity, safety perception, job satisfaction and overall quality of working life.

A key strength of the proposed methodology lies in its ability to operationalize a human-centered assessment grounded in physiological constraints, such as the maximum sustainable workload defined by NIOSH as approximately 33% of maximum voluntary contraction (71). In this sense, HURMAT extends beyond isolated or individualized risk analyses by enabling a multidimensional understanding of how environmental exposures and individual characteristics interact over time, shaping both the emergence and progression of occupational fatigue in real-world work settings.

### 5.4 Intervention and outcomes

Effective implementation of multilevel fatigue management interventions requires the active participation of all stakeholders throughout the entire process. The establishment of clear, well-communicated OHS policies plays a key role in addressing limitations in safety literacy and fostering worker engagement in improving workplace conditions (87–89). In this context, systematic fatigue management becomes not only a technical process but also an organizational and cultural commitment.

The HURMAT methodology does not merely operate as a monitoring tool; rather, it represents a shift in how occupational risks are conceptualized, managed and mitigated. Its implementation promotes a prevention-oriented culture grounded in personalization, data-driven decision-making and respect for individual variability, core principles for achieving sustainable safety outcomes in complex and continuously transforming work environments (90).

As demonstrated in the presented case study, the multilevel interventions guided by HURMAT had a clear positive impact on occupational outcomes. The worker-centered assessment and targeted intervention strategies contributed to improvements in musculoskeletal health, enhanced worker confidence and perceived safety, and measurable gains in productivity and work performance. These results indicate that systematic fatigue monitoring not only supports the prevention of occupational accidents and work-related diseases, but also positively influences productivity and work quality, directly addressing RQ4.

Moreover, the application of HURMAT enables the identification of environmental and task-related contributors to fatigue that increase incident risk when left unmanaged. For instance, in the analysed case study, exposure to extreme thermal conditions was shown to exacerbate fatigue through neuromuscular, physiological and metabolic mechanisms, accelerating fatigue onset. By combining real-time fatigue monitoring with multilevel intervention strategies, it becomes possible to define workload limits, adjust work rest cycles and apply preventive measures before fatigue translates into incidents or long-term health impairments.

The European Commission has increasingly emphasized fatigue management as a fundamental component of a robust safety culture, especially in the mobility and transport sectors, where fatigue-related performance degradation may have critical safety consequences. Fatigue management methodologies should not be designed or implemented in isolation or at an exclusively individual level; rather, they must be integrated in a holistic manner alongside other risk management approaches to effectively address the broad spectrum of hazards to which workers are exposed (91).

The concept of fatigue-proofing further reinforces this perspective by highlighting that, beyond managing the underlying causes of fatigue, greater emphasis should be placed on developing system-level strategies aimed at mitigating and reducing the impacts of fatigue on human performance and operational safety. This approach represents a shift from an individual-centered paradigm toward a proactive and systemic prevention strategy (35).

In addition to its administrative and real-time fatigue management components, the HURMAT framework can be conceptualized as incorporating engineering-level fatigue-proofing controls. These controls act at the system design level by modifying task allocation, automation levels, and operational constraints based on physiological indicators of operator state. This aligns with the principles of resilience engineering, where system performance is maintained through adaptive reconfiguration rather than solely relying on behavioral adaptation (92). Specifically, physiological data captured within HURMAT may trigger adaptive automation strategies, dynamic task redesign, and safety constraint activation, thereby reducing reliance on human performance under conditions of fatigue. This approach is consistent with human factors literature emphasizing error-tolerant system design and automation support in degraded performance states (93, 94).

### 5.5 Technological integration

The labor market is undergoing rapid reconfiguration driven by advanced digitalization and the integration of autonomous systems, demanding a fundamental reassessment of traditional work organization models. In this context, intelligent digital systems play a critical role in mitigating occupational risks by enhancing monitoring, control and incident prevention mechanisms, particularly in high-risk industrial environments. Consequently, this domain has been identified as a priority area by the European Agency for Safety and Health at Work (95).

Emerging technologies demonstrate the highest effectiveness in fatigue management when embedded within human-centered, multilevel frameworks, enabling proactive, adaptive and personalized interventions, rather than being deployed as isolated monitoring solutions. The use of wearables for continuous monitoring of workers and working environments is therefore essential for the early identification of critical and hazardous situations (85, 90). This integrated analysis of multiple factors allows for the identification of the most significant fatigue inducing risks and their impacts on workers’ health, supporting multifaceted preventive, improvement and mitigation interventions (82).

Within this context, the primary advantage of the HURMAT methodology lies in its capacity to adapt to diverse occupational environments, directly addressing RQ5. Rather than imposing a single, standardized approach, HURMAT is conceived as a flexible framework that can integrate complementary and emerging methodologies, enabling customization according to the specific work context under evaluation and continuous adjustment within a rapidly evolving technological environment. This adaptability supports the incorporation of diverse technologies for identifying fatigue related risk factors, alongside different approaches for continuous worker monitoring. Furthermore, the implementation of the HURMAT methodology provides structured decision support for stakeholders, facilitating continuous improvement of workplaces and contributing to the development of safer, healthier and more satisfying working conditions.

### 5.6 Limitations

Nevertheless, several limitations and implementation challenges may arise upon application of the proposed approach, particularly regarding worker acceptance and data management. The HURMAT requires the collection of personal data, work-related recordings, and clinical information, which may raise concerns among workers about privacy, as well as issues related to data management, ownership, and liability.

It is therefore imperative to ensure that all collected data complies with the General Data Protection Regulation and that workers provide informed consent for data collection. Strict adherence to privacy legislation is essential for the ethical implementation of monitoring systems. Organizations must remain up to date with applicable legal requirements, which entails regularly reviewing and updating privacy policies to align with current regulatory frameworks. Furthermore, establishing clear and well-communicated OHS policies within organizations can help address low levels of safety literacy and promote active worker involvement in improving workplace conditions. Companies should develop comprehensive data privacy policies that clearly define the methods and purposes of data collection, storage, and use. Clear communication ensures that workers understand the potential benefits of fatigue management tools and providing concise and accessible informational materials, complemented by training sessions, can enhance transparency, build trust, and improve worker acceptance.

Additionally, even though the proposed methodology includes adaptive fatigue management measures, specific engineering controls for fatigue-proofing were not explicitly developed in the present study. Future developments of the HURMAT framework should explore error-tolerant system design and other fatigue-proofing controls aimed at preventing fatigue-related errors from escalating into incidents.

Finally, this methodology is inherently complex, which may limit its practical application by OHS teams, as it requires a multidisciplinary assessment of both occupational parameters and contextual factors. Moreover, the use of monitoring equipment and advanced analytical approaches may generate additional costs, increasing OHS expenditures that organizations may be reluctant to assume. Despite this, the case study presented demonstrated that the approach is feasible to implement and provides benefits. This case study was intended to provide a first validation of the method, but further case studies will be required to validate the effectiveness of the HURMAT.

Beyond the case study presented, comparable multimodal fatigue management strategies have been implemented across multiple sectors and industries, including aviation, healthcare, safety, and logistics. In aviation, Bartulović and Grgić (13) reported the implementation of an aviation fatigue risk management system in multiple airlines. Such systems differ across countries and rely on a variety of specialized software and tools to monitor and mitigate fatigue-related risks in aviation. Despite the differences between systems, its implementation led to significant improvements in safety and operational efficiency, including a reduction in the subjective experience of fatigue among crew members (in some cases up to 93%), fewer fatigue-related incidents and operational errors (reductions ranging from 20 to 30%), and better roster management with more flexible work scheduling.

Healthcare is another field where there is evidence of the applicability of fatigue management systems. As an example, Dara developed a fatigue risk management system integrating roster analysis, reports on fatigue and sleep, actigraphy, vigilance testing, and situation-awareness assessments. Following implementation, clinicians showed reduced fatigue and sleepiness scores and improved situation awareness (96). Surgeries are other example of application in healthcare, where, for example, Merbah et al. demonstrated fatigue management during live surgery using wearable electromyography and posture sensors to quantify phase-dependent muscular fatigue, enabling identification of critical intraoperative periods where targeted interventions could be introduced (97).

Firefighting is other physical and mental demanding job which can benefit from fatigue management tools. Recent firefighter studies highlighted the need for real-time monitoring of physiological parameters to manage fatigue proactively and enhance both health and operational safety (98). Some examples of such fatigue management include the work from Billings et al., which assessed fatigue and recovery using wrist actigraphy (total sleep time, sleep efficiency, wake after sleep onset) alongside validated self-report instruments for insomnia, fatigue, and mental health. The intervention resulted in increases in total sleep time and sleep efficiency, accompanied by reductions in insomnia and depressive symptom scores at 3 months, with benefits maintained at six-month follow-up (99). Complementing this organizational approach, Xu et al. provided a task-level, real-time fatigue management using physiological sensing and predictive modeling during firefighting activities. Fatigue was assessed using electrocardiogram signals, heart-rate variability metrics, reaction-time tests, and perceived exertion scales, and a multi-level fatigue classification model was developed and integrated with a machine-learning predictor to differentiate light, moderate, and severe fatigue states (100).

In mining operations, multimodal fatigue management strategies combining organizational, technological, and individual-level data have been implemented. For example, Pan et al. (101) proposed a multimodal AI system to detect fatigue in miners based on physiological data (electrocardiogram, electrodermal activity, blood pressure, blood oxygen saturation, skin temperature, etc.). By combining multiple data sources, it classifies fatigue states more accurately, improving the reliability of fatigue monitoring systems in real-world mining conditions (101). Similarly, Rogers et al. proposed an IoT-based wearable system for monitoring miner fatigue using both subjective self-reports and objective measures. By combining multiple inputs, the system provided a more continuous and accurate assessment of fatigue than traditional methods, supporting decision-making (102).

Construction is also a field where workers face multiple musculoskeletal disorders and there is recent literature exploring the use of fatigue management systems to reduce them. For instance, Tao et al. (103) proposed a two-hierarchy method to assess the ergonomic risk, considering both postural and non-postural factors. A real-life case study was conducted, which demonstrated its applicability and effectiveness (103). Another study developed a mixed-integer linear programming approach to optimize the work–rest schedule by integrating workers’ overexertion based on worker and task information. The approach was validated in a case study, and the results suggested that up to 20% improvement in productive time and a reduction in occupational risks resulting from overexertion (104).

These case studies show that combining physiological, behavioral, and organizational measures is feasible and effective across fields, reinforcing the generalizability of the proposed methodology beyond the single case study presented. Together, the examples further support the proposed methodology’s potential to improve both safety and productivity across different operational contexts.

## 6 Conclusion

In a constantly evolving work environment, driven by technological innovation and the emergence of new forms of work organization, OHS teams must adapt their practices to keep pace with evolving production processes while ensuring the protection of workers’ health and safety. However, traditional risk assessment and control methodologies are often insufficient to address the complex and dynamic demands of contemporary work settings, thereby limiting their effectiveness in mitigating occupational risks.

This context underscores the urgent need for the development and implementation of innovative approaches that respond to the ongoing transformations in the workplace. Such approaches should not only focus on working conditions but also consider the worker’s functional capacity in relation to task demands. The HURMAT methodology represents an innovative proposal in this regard, integrating the core principles of traditional methods with a more in-depth, worker-centered evaluation of functional capacity. As such, it offers a valuable contribution to the modernization of OHS practices and the promotion of safer and more adaptive work environments.

## Data Availability

The original contributions presented in the study are included in the article/supplementary material, further inquiries can be directed to the corresponding authors.

## References

[1] European Commission, Eurostat. Accidents at Work Statistics. Luxembourg: Publications Office of the European Union (2024). Disponível em: Available online at: https://ec.europa.eu/eurostat/statistics-explained/index.php?title=Accidents_at_work_statistics.

[2] Akyildiz C. Integration of digitalization into occupational health and safety and its applicability: a literature review. Eur Res J. (2023) 9:1509–19. doi: 10.18621/eurj.1352743

[3] Çalış S Büyükakıncı BY. Occupational health and safety management systems applications and a system planning model. Procedia Comput Sci. (2019) 158:1058–66. doi: 10.1016/j.procs.2019.09.147

[4] Kineber AF Antwi-Afari MF Elghaish F Zamil AMA Alhusban M Qaralleh TJO. Benefits of implementing occupational health and safety Management Systems for the Sustainable Construction Industry: a systematic literature review. Sustainability. (2023) 15:12697. doi: 10.3390/su151712697

[5] Alli BO. Fundamental Principles of Occupational Health and Safety. 2nd ed. (2008). Geneva: International Labour Office (ILO).

[6] ILO. (2016). Occupational Safety and Health Management System. Geneva: International Labour Office.

[7] European Agency for Safety and Health at Work (EU-OSHA). Advanced Robotics and Automation: Implications for Occupational Safety and Health. Bilbao: EU-OSHA (2022).

[8] Chander DS Cavatorta MP. An observational method for postural ergonomic risk assessment (PERA). Int J Ind Ergon. (2017) 57:32–41. doi: 10.1016/j.ergon.2016.11.007

[9] Castellucci HI Viviani C Hernández P Bravo G Martínez M Ibacache J . Developing countries and the use of ISO standard 11228-3 for risk management of work-related musculoskeletal disorders of the upper limbs (WRMSDs-ULs): the case of Chile. Appl Ergon. (2021) 96:103483. doi: 10.1016/j.apergo.2021.103483, 34102576

[10] Beltran Martinez K Nazarahari M Rouhani H. K-score: a novel scoring system to quantify fatigue-related ergonomic risk based on joint angle measurements via wearable inertial measurement units. Appl Ergon. (2022) 102:103757. doi: 10.1016/j.apergo.2022.103757, 35378482

[11] Canadian Centre for Occupational Health and Safety (CCOHS). Fatigue. Hamilton, ON: CCOHS. Available online at (2024). (https://www.ccohs.ca/oshanswers/psychosocial/fatigue.html

[12] Cunningham TR Guerin RJ Ferguson J Cavallari J. Work-related fatigue: a hazard for workers experiencing disproportionate occupational risks. Am J Ind Med. (2022) 65:913–25. doi: 10.1002/ajim.23325, 35088430 PMC9325913

[13] Bartulović D Grgić A. Implementing fatigue risk management system in an airline: a case study. Transp Res Procedia. (2025) 91:13–20. doi: 10.1016/j.trpro.2025.10.003

[14] Bendak S Rashid HSJ. Fatigue in aviation: a systematic review of the literature. Int J Ind Ergon. (2020) 76:102928. doi: 10.1016/j.ergon.2020.102928

[15] Wong I Swanson N. Approaches to managing work-related fatigue to meet the needs of American workers and employers. Am J Ind Med. (2022) 65:827–31. doi: 10.1002/ajim.23402, 35661203 PMC10583120

[16] Fitsilis P. Tsoutsa P. Damasiotis V. Kyriatzis V. (2024). Uncovering key trends in industry 5.0 through advanced AI techniques.

[17] Arana-Landín G Laskurain-Iturbe I Iturrate M Landeta-Manzano B. Assessing the influence of industry 4.0 technologies on occupational health and safety. Heliyon. (2023) 9:e13720. doi: 10.1016/j.heliyon.2023.e13720, 36950597 PMC10025012

[18] Dodoo JE Al-Samarraie H Alzahrani AI Lonsdale M Alalwan N. Digital innovations for occupational safety: empowering workers in hazardous environments. Workplace Health Safety. (2024) 72:84–95. doi: 10.1177/21650799231215811, 38193448 PMC10928957

[19] Bęś P Strzałkowski P Górniak-Zimroz J Szóstak M Janiszewski M. Innovative technologies to improve occupational safety in mining and construction industries—part I. Sensors. (2025) 25:5201. doi: 10.3390/s25165201, 40872063 PMC12390005

[20] Hanna A Larsson S Götvall P-L Bengtsson K. Deliberative safety for industrial intelligent human–robot collaboration: regulatory challenges and solutions for taking the next step towards industry 4.0. Robot Comput Integr Manuf. (2022) 78:102386. doi: 10.1016/j.rcim.2022.102386

[21] Leng J Sha W Wang B Zheng P Zhuang C Liu Q . Industry 5.0: Prospect and retrospect. J Manuf Syst. (2022) 65:279–95. doi: 10.1016/j.jmsy.2022.09.017

[22] Leso V Fontana L Iavicoli I. The occupational health and safety dimension of industry 4.0. Med Lav. (2018) 110:327–38. doi: 10.23749/mdl.v110i5.7282, 30378585 PMC7682172

[23] Meindl B Ayala NF Mendonça J Frank AG. The four smarts of industry 4.0: evolution of ten years of research and future perspectives. Technol Forecast Soc Change. (2021) 168:120784. doi: 10.1016/j.techfore.2021.120784

[24] Robla-Gomez S Becerra VM Llata JR Gonzalez-Sarabia E Torre-Ferrero C Perez-Oria J. Working together: a review on safe human-robot collaboration in industrial environments. IEEE Access. (2017) 5:26754–73. doi: 10.1109/ACCESS.2017.2773127

[25] Caggiano A Grant R Peng C Li Z Simeone A. Manufacturing process impacts on occupational health: a machine learning framework. Procedia CIRP. (2022) 112:561–6. doi: 10.1016/j.procir.2022.09.100

[26] Milea A Cioca LI. Work evolution and safety and health at work in industry 4.0/industry 5.0. MATEC Web Conf. (2024) 389:00074. doi: 10.1051/matecconf/202438900074

[27] Gómez-Galán M Pérez-Alonso J Callejón-Ferre Á-J López-Martínez J. Musculoskeletal disorders: OWAS review. Ind Health. (2017) 55:314–37. doi: 10.2486/indhealth.2016-0191, 28484144 PMC5546841

[28] Lowe BD Dempsey PG Jones EM. Ergonomics assessment methods used by ergonomics professionals. Appl Ergon. (2019) 81:102882. doi: 10.1016/j.apergo.2019.102882 31422255

[29] Avgoustaki A Frankort HTW. Implications of work effort and discretion for employee well-being and career-related outcomes: an integrative assessment. ILR Rev. (2019) 72:636–61. doi: 10.1177/0019793918804540

[30] Eurofound. *European Working Conditions Telephone Survey 2021*. Dublin: European Foundation for the Improvement of Living and Working Conditions. (2023). Available at: https://www.eurofound.europa.eu/en/data-catalogue/european-working-conditions-telephone-survey-2021-0

[31] Van Iddekinge CH Arnold JD Aguinis H Lang JWB Lievens F. Work effort: a conceptual and meta-analytic review. J Manage. (2023) 49:125–57. doi: 10.1177/01492063221087641

[32] Adão Martins NR Annaheim S Spengler CM Rossi RM. Fatigue monitoring through wearables: a state-of-the-art review. Front Physiol. (2021) 12:790292. doi: 10.3389/fphys.2021.790292, 34975541 PMC8715033

[33] Chen Y Li S Kuang J Zhang X Zhou Z Li E-J . Biomechanical monitoring of exercise fatigue using wearable devices: a review. Bioengineering. (2025) 13:13. doi: 10.3390/bioengineering13010013, 41595945 PMC12838368

[34] Kakhi K Jagatheesaperumal SK Khosravi A Alizadehsani R Acharya UR. Fatigue monitoring using wearables and AI: trends, challenges, and future opportunities. Comput Biol Med. (2025) 195:110461. doi: 10.1016/j.compbiomed.2025.110461, 40580618

[35] Dawson D Chapman J Thomas MJW. Fatigue-proofing: a new approach to reducing fatigue-related risk using the principles of error management. Sleep Med Rev. (2012) 16:167–75. doi: 10.1016/j.smrv.2011.05.004, 21784677

[36] Vilisov VY Dyatlova DA. Analysis of Information Technologies Used to Insure Working Efficiency of Personnel. Resources and Regions Effective Use (2016). Available online at: https://arxiv.org/pdf/1705.03507v1.pdf

[37] MassirisFernández M Fernández JÁ Bajo JM Delrieux CA. Ergonomic risk assessment based on computer vision and machine learning. Comput Ind Eng. (2020) 149:106816. doi: 10.1016/j.cie.2020.106816

[38] MacIntosh BR Murias JM Keir DA Weir JM. What is moderate to vigorous exercise intensity? Front Physiol. (2021) 12:2233. doi: 10.3389/fphys.2021.682233, 34630133 PMC8493117

[39] Kirci BK Ensari Ozay M Ucan R. A case study in ergonomics by using REBA, RULA and NIOSH methods: logistics warehouse sector in Turkey. Hittite J. Sci. Eng. (2020) 7:257–64. doi: 10.17350/HJSE19030000194

[40] Korshøj M Lund Rasmussen C de Oliveira Sato T Holtermann A Hallman D. Heart rate during work and heart rate variability during the following night: a day-by-day investigation on the physical activity paradox among blue-collar workers. Scand J Work Environ Health. (2021) 47:387–94. doi: 10.5271/sjweh.3965, 33929548 PMC8259705

[41] Krause N Brand RJ Kaplan GA Kauhanen J Malla S Tuomainen T-P . Occupational physical activity, energy expenditure and 11-year progression of carotid atherosclerosis. Scand J Work Environ Health. (2007) 33:405–24. doi: 10.5271/sjweh.1171, 18327509

[42] Schettino S Minette LJ Andrade Lima RC Pedroso Nascimento GS Caçador SS Leme Vieira MP. Forest harvesting in rural properties: risks and worsening to the worker’s health under the ergonomics approach. Int J Ind Ergon. (2021) 82:103087. doi: 10.1016/j.ergon.2021.103087

[43] Pichot V Bourin E Roche F Garet M Gaspoz J-M Duverney D . Quantification of cumulated physical fatigue at the workplace. Eur J Phys. (2002) 445:267–72. doi: 10.1007/s00424-002-0917-7, 12457247

[44] Anwer S Li H Umer W Antwi-Afari MF Mehmood I Yu Y . Identification and classification of physical fatigue in construction workers using linear and nonlinear heart rate variability measurements. J. Construct. Eng. Manage. (2022) 149. doi: 10.1061/JCEMD4.COENG-13100

[45] Grossman P Taylor EW. Toward understanding respiratory sinus arrhythmia: relations to cardiac vagal tone, evolution and biobehavioral functions. Biol Psychol. (2007) 74:263–85. doi: 10.1016/j.biopsycho.2005.11.014, 17081672

[46] Thayer JF Yamamoto SS Brosschot JF. The relationship of autonomic imbalance, heart rate variability and cardiovascular disease risk factors. Int J Cardiol. (2010) 141:122–31. doi: 10.1016/j.ijcard.2009.09.543, 19910061

[47] Yoopat P Pitakwong P Vanwonterghem K. Assessing the physiological strain of physical therapists according to work experience: a cross-sectional study. J Bodyw Mov Ther. (2020) 24:253–62. doi: 10.1016/j.jbmt.2019.05.033, 31987554

[48] Trougakos JP Hideg I. Momentary work recovery: the role of within-day work breaks. Res. Occup. Stress Well Being. (2009) 7:37–84. doi: 10.1108/S1479-3555(2009)0000007005

[49] Trougakos JP Hideg I Cheng BH Beal DJ. Lunch breaks unpacked: the role of autonomy as a moderator of recovery during lunch. Acad Manag J. (2014) 57:405–21. doi: 10.5465/amj.2011.1072

[50] Tucker P. The impact of rest breaks upon accident risk, fatigue and performance: a review. Work Stress. (2003) 17:123–37. doi: 10.1080/0267837031000155949

[51] Zijlstra FRH Cropley M Rydstedt LW. From recovery to regulation: an attempt to reconceptualize ‘recovery from work.’. Stress Health. (2014) 30:244–52. doi: 10.1002/smi.2604 25100275

[52] International Organization for Standardization (ISO). ISO 45001:2018 - Occupational Health and Safety Management Systems — Requirements with Guidance for Use. Geneva: ISO (2018).

[53] International Organization for Standardization (ISO). ISO 31000:2018 Risk management — Guidelines. Geneva: ISO (2018).

[54] Sena IP Braun J Pereira AI. “Data analysis of workplace accidents – a case study”. In: Optimization, Learning Algorithms and Applications Communications in Computer and Information Science. Cham: Springer (2021). 571–86.

[55] Thallapureddy S Sherratt F Hallowell M Bhandari S. Effective information collection in incident investigations: a systematic review and narrative synthesis. Saf Sci. (2024) 171:106404. doi: 10.1016/j.ssci.2023.106404

[56] Gotardelo MPS Rodrigues ALM Quaresma FRP Pontes-Silva A Maciel E d S. Work-related musculoskeletal disorders in vulnerable populations: what are the most common body parts affected? BMC Public Health. (2023) 23:1635. doi: 10.1186/s12889-023-16570-2, 37626280 PMC10464016

[57] Rantala M Lindholm M Tappura S. Supporting occupational health and safety risk assessment skills: a case study of five companies. Int J Environ Res Public Health. (2022) 19:1720. doi: 10.3390/ijerph19031720, 35162743 PMC8835380

[58] Saravi B Kabirzadeh A Rezazadeh E Khariki M Zolaykha Z Asgari A . Prevalence and causes of medical absenteeism among staff (case study at Mazandaran University of Medical Sciences: 2009-2010). Mater Soc Med. (2013) 25:233–7. doi: 10.5455/msm.2013.25.233-237, 24511264 PMC3914745

[59] Khoe LC Saldi SRF Isbayuputra M Mansyur M Wiseman V Asante A. Global trends in occupational disease reporting: a systematic review. medRxiv. (2024). doi: 10.1101/2024.09.19.24314032

[60] Denis D Lortie M Rossignol M. Observation procedures characterizing occupational physical activities: critical review. Int J Occup Saf Ergon. (2000) 6:463–91. doi: 10.1080/10803548.2000.11076467, 11135680

[61] Yngve M Ekbladh E. Clinical utility of the worker role interview: a survey study among Swedish users. Scand J Occup Ther. (2015) 22:416–23. doi: 10.3109/11038128.2015.1007161, 25797368

[62] Heng PP Mohd Yusoff H Hod R. Individual evaluation of fatigue at work to enhance the safety performance in the construction industry: a systematic review. PLoS One. (2024) 19:e0287892. doi: 10.1371/journal.pone.0287892, 38324557 PMC10849240

[63] Masci F Spatari G Bortolotti S Giorgianni CM Antonangeli LM Rosecrance J . Assessing the impact of work activities on the physiological load in a sample of loggers in Sicily (Italy). Int J Environ Res Public Health. (2022) 19:7695. doi: 10.3390/ijerph19137695, 35805360 PMC9265621

[64] Menychtas D Glushkova A Manitsaris S. Analyzing the kinematic and kinetic contributions of the human upper body’s joints for ergonomics assessment. J Ambient Intell Humaniz Comput. (2020) 11:6093–105. doi: 10.1007/s12652-020-01926-y

[65] Clusiault D Avery T Stephens A Vigna C Fischer SL. Scoping review on the state of the integration of human physiological responses to evaluating heat-stress. Appl Ergon. (2022) 101:103704. doi: 10.1016/j.apergo.2022.103704, 35139444

[66] Ioannou LG Foster J Morris NB Piil JF Havenith G Mekjavic IB . Occupational heat strain in outdoor workers: a comprehensive review and meta-analysis. Temperature. (2022) 9:67–102. doi: 10.1080/23328940.2022.2030634, 35655665 PMC9154804

[67] Sollers JJ Sanford TA Nabors-Oberg R Anderson CA Thayer JF. Examining changes in HRV in response to varying ambient temperature. IEEE Eng Med Biol Mag. (2002) 21:30–4. doi: 10.1109/MEMB.2002.1032636, 12222114

[68] Mahdavi N Tapak L Darvishi E Doosti-Irani A Shafiee Motlagh M. Unraveling the interplay between mental workload, occupational fatigue, physiological responses and cognitive performance in office workers. Sci Rep. (2024) 14:17866. doi: 10.1038/s41598-024-68889-4, 39090219 PMC11294527

[69] Koutsimani P Montgomery A Georganta K. The relationship between burnout, depression, and anxiety: a systematic review and Meta-analysis. Front Psychol. (2019) 10:284. doi: 10.3389/fpsyg.2019.00284, 30918490 PMC6424886

[70] Lari M. A longitudinal study on the impact of occupational health and safety practices on employee productivity. Saf Sci. (2024) 170:106374. doi: 10.1016/j.ssci.2023.106374

[71] National Institute for Occupational Safety and Health (NIOSH). Work Practices Guide for Manual Lifting. Cincinnati, OH: NIOSH (1981).

[72] International Organization for Standardization (ISO). ISO 8996:2004 - Ergonomics of the Thermal Environment — Determination of Metabolic Rate. Geneva: ISO (2004).

[73] Vitrano G Urso D Micheli GJL Guglielmi A De Merich D Pellicci M. Enabling effective implementation of occupational safety and health interventions. Saf Health Work. (2024) 15:213–9. doi: 10.1016/j.shaw.2024.04.003, 39035805 PMC11255946

[74] Armstrong TJ. *Job/Task Analysis Fundamentals*. Ann Arbor, MI: University of Michigan. (2023). Available online at: http://www-personal.umich.edu/~tja/task/taskanal.html.

[75] International Organization for Standardization (ISO). ISO 11228-2:2007 - Ergonomics — Manual Handling — Part 2: Pushing and Pulling. Geneva: ISO (2007).

[76] International Organization for Standardization (ISO). ISO 11228-3:2007 - Ergonomics — Manual Handling — Part 3: Handling of Low Loads at High Frequency. Geneva: ISO (2007).

[77] International Organization for Standardization (ISO). ISO/TR 12295:2014 - Ergonomics — Application Document for International Standards on Manual Handling (ISO 11228-1, ISO 11228-2 and ISO 11228-3) and Evaluation of Static Working Postures (ISO 11226). Geneva: ISO (2014).

[78] de Weerd M Tierney R van Duuren-Stuurman B Bertranou E. Estimating the Cost of Accidents and Ill-Health at Work: A Review of Methodologies. Luxembourg: Publications Office of the European Union (2014).

[79] Ouyang Y Liu M Cheng C Yang Y He S Zheng L. Monitoring inattention in construction workers caused by physical fatigue using electrocardiograph (ECG) and galvanic skin response (GSR) sensors. Sensors (Basel). (2023) 23:7405. doi: 10.3390/s23177405, 37687860 PMC10490619

[80] Pütz S Mertens A Chuang LL Nitsch V. Physiological predictors of operator performance: the role of mental effort and its link to task performance. Hum Factors. (2024) 67:6830. doi: 10.1177/00187208241296830, 39477348 PMC12049591

[81] Banani Ardecani F Shoghli O. Assessing workers’ neuro-physiological stress responses to augmented reality safety warnings in immersive virtual roadway work zones. Autom Constr. (2025) 180:106565. doi: 10.1016/j.autcon.2025.106565

[82] Soto L Ron M. Use of physiological indicators as determinants of physical aptitude in work environments in Latin America: a systematic review. Rehabil Sports Med. (2025) 5:179. doi: 10.56294/ri2025179

[83] Argyle EM Marinescu A Wilson ML Lawson G Sharples S. Physiological indicators of task demand, fatigue, and cognition in future digital manufacturing environments. Int J Hum Comput Stud. (2021) 145:102522. doi: 10.1016/j.ijhcs.2020.102522

[84] Li M Ma Z Yan R Yin J. The impact of physiological and psychological fatigue on work efficiency: a case study of parcel sorting work. Sensors. (2024) 24:5989. doi: 10.3390/s24185989, 39338736 PMC11435520

[85] Mohanavelu K Lamshe R Poonguzhali S Adalarasu K Jagannath M. Assessment of human fatigue during physical performance using physiological signals: a review. Biomed Pharmacol J. (2017) 10:1887–96. doi: 10.13005/bpj/1308

[86] Cham BS Boeing AA Wilson MK Griffin MA Jorritsma K. Endurance in extreme work environments. Organ Psychol Rev. (2021) 11:343–64. doi: 10.1177/20413866211006441

[87] Ehmann AT Ög E Rieger MA Siegel A. Work-related health literacy: a scoping review to clarify the concept. Int J Environ Res Public Health. (2021) 18:9945. doi: 10.3390/ijerph18199945, 34639262 PMC8507793

[88] Pejtersen JH Holt H. Literacy and risk of occupational injury. Int Arch Occup Environ Health. (2022) 95:1971–8. doi: 10.1007/s00420-022-01898-w, 35748939

[89] Torun SD. Occupational health literacy level and related factors in casting factory workers. Bakirkoy Tip Dergisi/Med J Bakirkoy. (2023) 19:229–35. doi: 10.4274/BMJ.galenos.2023.2023.4-6

[90] European Commission. (2026). *Fatigue Management Programs*. Brussels: European Commission. Available online at: (https://road-safety.transport.ec.europa.eu/european-road-safety-observatory/statistics-and-analysis-archive/fatigue/fatigue-management-programs_en).

[91] European Commission (2026). Fatigue Management Programs. Available online at: https://road-safety.transport.ec.europa.eu/european-road-safety-observatory/statistics-and-analysis-archive/fatigue/fatigue-management-programs_en

[92] Hollnagel E. Safety–I and Safety–II: The Past and Future of Safety Management. Boca Raton, FL: CRC Press (2018).

[93] Lee JD Seppelt BD. "Human factors in automation design". In: Springer Handbook of Automation. Berlin Heidelberg: Springer (2009). p. 417–36.

[94] Reason J. Managing the Risks of Organizational Accidents. London: Routledge (2016).

[95] European Agency for Safety and Health at Work (EU-OSHA). Strategies for Safety and Health in an Automated World. Bilbao: EU-OSHA (2024).

[96] Dara S. Impact of fatigue risk management system on fatigue and situation awareness of surgical intensive care unit nurses. Intern Med J. (2019) 49:19. doi: 10.1111/imj.4_14299

[97] Merbah J Caré BR Gorce P Gadea F Prince F. A new approach to quantifying muscular fatigue using wearable EMG sensors during surgery: an ergonomic case study. Sensors. (2023) 23:1686. doi: 10.3390/s23031686, 36772729 PMC9919042

[98] Teixeira T Pratas P Santos J Monteiro PR Baptista JS Vaz MAP . Physical demand assessment of volunteer firefighters during wildland firefighting. Fire. (2024) 7:439. doi: 10.3390/fire7120439

[99] Billings JM Jahnke SA. Effects of a 24/48 to 48/96 shift schedule change on firefighter sleep and health: short-term improvements and six-month stability. Int J Environ Res Public Health. (2025) 22:1678. doi: 10.3390/ijerph22111678, 41302624 PMC12652382

[100] Xu M Yang S Wang K Yu C Liu G Dai C . A study on the classification and prediction of firefighter’s operational fatigue level. PLoS One. (2025) 20:e0323911. doi: 10.1371/journal.pone.0323911, 40373073 PMC12080770

[101] Pan H Tong S Wei X Teng B. Fatigue state recognition system for miners based on a multimodal feature extraction and fusion framework. IEEE Trans. Cogn. Dev. Syst. (2025) 17:410–20. doi: 10.1109/TCDS.2024.3461713

[102] Rogers WP Marques J Talebi E Drews FA. IoT-enabled wearable fatigue-tracking system for mine operators. Minerals. (2023) 13:287. doi: 10.3390/min13020287

[103] Tao Y Hu H Xu F Zhang Z. Ergonomic risk assessment of construction workers and projects based on fuzzy Bayesian network and D-S evidence theory. J Constr Eng Manag. (2023) 149:2821. doi: 10.1061/JCEMD4.COENG-12821

[104] Tao Y Hu H Xu F Zhang Z. Work–rest schedule optimization of precast production considering workers’ overexertion. J Constr Eng Manag. (2024) 150:4377. doi: 10.1061/JCEMD4.COENG-14377

